# HOMA-IR Values are Associated With Glycemic Control in Japanese Subjects Without Diabetes or Obesity: The KOBE Study

**DOI:** 10.2188/jea.JE20140172

**Published:** 2015-06-05

**Authors:** Takumi Hirata, Aya Higashiyama, Yoshimi Kubota, Kunihiro Nishimura, Daisuke Sugiyama, Aya Kadota, Yoko Nishida, Hironori Imano, Tomofumi Nishikawa, Naomi Miyamatsu, Yoshihiro Miyamoto, Tomonori Okamura

**Affiliations:** 1 Foundation for Biomedical Research and Innovation, Kobe, Japan; 1 公益財団法人 先端医療振興財団; 2 Department of Preventive Medicine and Epidemiologic Informatics, National Cerebral and Cardiovascular Center, Osaka, Japan; 2 国立循環器病研究センター 予防医学・疫学情報部; 3 Department of Environmental and Preventive Medicine, Hyogo College of Medicine, Nishinomiya, Hyogo, Japan; 3 兵庫医科大学 環境予防医学; 4 Department of Preventive Medicine and Public Health, School of Medicine, Keio University, Tokyo, Japan; 4 慶應義塾大学医学部 衛生学公衆衛生学; 5 Department of Public Health, Shiga University of Medical Science, Ohtsu, Japan; 5 滋賀医科大学 アジア疫学研究センター; 6 Department of Social Medicine, Graduate School of Medicine, Osaka University, Suita, Osaka, Japan; 6 大阪大学大学院医学系研究科 公衆衛生学; 7 Faculty of Health Science, Kyoto Koka Women’s University, Kyoto, Japan; 7 京都光華女子大学 健康科学部; 8 Department of Clinical Nursing, Shiga University of Medical Science, Ohtsu, Japan; 8 滋賀医科大学 臨床看護学講座

**Keywords:** homeostasis model assessment of insulin resistance, glycemic control, epidemiology

## Abstract

**Background:**

Several studies have reported that insulin resistance was a major risk factor for the onset of type 2 diabetes mellitus in individuals without diabetes or obesity. We aimed to clarify the association between insulin resistance and glycemic control in Japanese subjects without diabetes or obesity.

**Methods:**

We conducted a community-based cross-sectional study including 1083 healthy subjects (323 men and 760 women) in an urban area. We performed multivariate regression analyses to estimate the association between the homeostasis model assessment of insulin resistance (HOMA-IR) values and markers of glycemic control, including glycated haemoglobin (HbA1c), 1,5-anhydroglucitol (1,5-AG), and fasting plasma glucose (FPG) levels, after adjustment for potential confounders.

**Results:**

Compared with the lowest tertile of HOMA-IR values, the highest tertile was significantly associated with HbA1c and FPG levels after adjustment for potential confounders, both in men (HbA1c: β = 1.83, *P* = 0.001; FPG: β = 0.49, *P* < 0.001) and women (HbA1c: β = 0.82, *P* = 0.008; FPG: β = 0.39, *P* < 0.001). The highest tertile of HOMA-IR values was inversely associated with 1,5-AG levels compared with the lowest tertile (β = −18.42, *P* = 0.009) only in men.

**Conclusions:**

HOMA-IR values were associated with markers of glycemic control in Japanese subjects without diabetes or obesity. Insulin resistance may influence glycemic control even in a lean, non-diabetic Asian population.

## Abstract (ja)

**【背景】:**

これまで肥満ではなく糖尿病も有さない健常者においてインスリン抵抗性は2型糖尿病の危険因子であることが報告されているが、肥満度の低い日本人集団では検証されていない。そこで肥満・糖尿病を有さない日本人において、インスリン抵抗性が血糖コントロール指標と関連するかについて検討した。

**【方法】:**

都市住民の健常者1,083名（男性323名、女性760名、平均BMI 21.5 kg/m^2^）のデータを用いて断面研究を行った。交絡因子調整後におけるインスリン抵抗性の指標（HOMA-IR）と血糖コントロール指標（HbA1c、1,5-AG、空腹時血糖）の関連について、多変量回帰分析で検討した。

**【結果】:**

交絡因子調整後の結果において、男女ともHOMA-IR最低三分位群と比較し、最高三分位群でHbA1cおよび空腹時血糖が有意に高かった（男性: HbA1c; β = 1.83, *P* = 0.001, 空腹時血糖; β = 0.49, *P* < 0.001、女性: HbA1c; β = 0.82, *P* = 0.008, 空腹時血糖; β = 0.39, *P* < 0.001）。また男性でのみ、HOMA-IR最低三分位群と比較し、最高三分位群で1,5-AGが有意に低かった（β = −18.42, *P* = 0.009）。

**【結論】:**

肥満・糖尿病を有さない日本人においてHOMA-IRは血糖コントロール指標と関連しており、肥満や糖尿病を有さなくてもインスリン抵抗性が血糖コントロールに影響を及ぼしている可能性が示唆された。

## INTRODUCTION

Insulin resistance is a clinical condition characterized by a decreased sensitivity to insulin in peripheral tissues and is strongly associated with metabolic diseases, such as type 2 diabetes mellitus and obesity.^1^^–^^3^ Prospective cohort studies in subjects without diabetes have also revealed that increased insulin resistance worsened glycemic control and contributed to the development of type 2 diabetes mellitus.^4^^–^^6^ However, in all previous reports, the average body mass index (BMI) of the subjects was high (28–33 kg/m^2^), and >50% of the subjects were obese.^4^^–^^6^ Thus, it is unclear whether insulin resistance affects glycemic control in subjects without obesity or diabetes.

For assessing glycemic control, temporal variations in the indicative parameters are more important than values obtained at a single point in time. Glycated hemoglobin (HbA1c) and 1,5-anhydroglucitol (1,5-AG) levels are generally used to evaluate glycemic control in clinical practice. HbA1c levels are the gold standard marker of glycemic control in patients with diabetes, and they reflect average plasma glucose levels during the past 2–3 months.^7^ In contrast, 1,5-AG levels are used as an index that reflects glycemic control during the past few days or weeks and glycemic control fluctuations.^8^^,^^9^ Thus, it is necessary to use several indices in various time periods to evaluate glucose metabolism. However, to the best of our knowledge, no earlier studies have investigated the association between insulin resistance and glucose metabolism using multiple markers of glycemic control.

Therefore, in the present study, we aimed to investigate the impact of insulin resistance on glucose metabolism of Japanese subjects without diabetes or obesity. We used three markers that are commonly used to evaluate glucose metabolism in Japanese populations: HbA1c, 1,5-AG, and fasting plasma glucose (FPG) levels.

## METHODS

### Subjects

We used data from the baseline survey in the Kobe Orthopedic and Biomedical Epidemiological (KOBE) study. The KOBE study is a population-based prospective cohort study of risk factors for cardiovascular disease or worsening of quality of life in Kobe City, a major urban area in Japan, that has been ongoing since 2010. The KOBE study has been described in detail elsewhere.^10^ The present study was approved by the Ethics Committee of the Institute of Biomedical Research and Innovation (Committee approval number: 11-12). Written informed consent was obtained from all participants.

A total of 1118 subjects (342 men and 776 women) participated in the baseline survey from July 2010 to December 2011. None of the participants had past history of cardiovascular disease or cancer, and none were under therapy with medications for hypertension, dyslipidemia, or diabetes at the time of the survey. We excluded 34 participants who were diagnosed with diabetes or obesity on the basis of FPG level of ≥7.0 mmol/L (*n* = 8) and/or HbA1c level of ≥6.5% (*n* = 22) or BMI of ≥30 kg/m^2^ (*n* = 4) at baseline. A participant with missing data (*n* = 1) was also excluded. We ultimately analysed data of 1083 subjects (323 men and 760 women) without diabetes or obesity in this study.

### Measurements

Each subject completed a self-reported questionnaire to assess past medical history and lifestyle factors, such as smoking status, alcohol consumption, and regular exercise habits, and trained researchers directly confirmed the responses to the questionnaire. Waist circumference was measured at the level of the umbilicus in a standing position. Height and body weight were measured with patients wearing socks and light clothing, and BMI was calculated by dividing weight in kilograms by the squared height in meters.

Fasting blood samples were drawn from all participants after they had fasted for at least 10 hours. Blood samples were transported to a single commissioned clinical laboratory centre (SRL Inc., Tokyo, Japan) for measurements. Plasma glucose levels (mmol/L) were determined using the glucose oxidase method. 1,5-AG levels were measured using an enzymatic method. HbA1c levels were measured using high-performance liquid chromatography and were expressed as National Glycohemoglobin Standardization Program units and International Federation of Clinical Chemistry and Laboratory Medicine values for the current analysis.^11^ Serum immunoreactive insulin (IRI) levels (pmol/L) were determined using the chemiluminescence enzyme immunoassay (CLEIA) method, and homeostasis model assessment-insulin resistance (HOMA-IR) values were calculated using the following formula: HOMA-IR = IRI × glucose/22.5.^12^ Estimated glomerular filtration rate (eGFR) was calculated using the following formula: eGFR (mL/min per 1.73 m^2^) = 194 × creatinine^−1.094^ × age^−0.287^ (× 0.739 if female),^13^ and chronic kidney disease (CKD) defined as eGFR of <60 mL/min per 1.73 m^2^. High-molecular-weight adiponectin (HMW-adiponectin) levels were measured using the CLEIA method.

### Statistical analysis

Gender-specific analyses were performed in light of observed gender differences in HOMA-IR distribution. HOMA-IR values were divided into tertiles to compare the characteristics. Data were presented as means (standard deviations [SDs]) or medians (interquartile ranges) for continuous variables, or numbers (percentages) for categorical variables. We used one-way analysis of variance for continuous variables and the chi-square test or Fisher’s exact test for categorical variables to compare the characteristics among the groups. Multiple adjustments were performed with linear regression models to estimate the association between HOMA-IR values and markers of glycemic control, such as FPG, 1,5-AG, and HbA1c levels. We also performed multivariate logistic regression analysis to clarify the association between HOMA-IR values and any of the higher percentiles (80th or 90th percentile) of HbA1c levels, lower percentiles (10th or 20th percentile) of 1,5-AG levels or higher percentiles (80th or 90th percentile) of FPG levels. Multivariable analyses were adjusted for potential confounders in the following steps: (1) age; (2) BMI, regular exercise habits, current smoking, current alcohol drinking, CKD, and HMW-adiponectin levels, in addition to the variables in step 1; and (3) waist circumference substituted for BMI in step 2. The adjusted coefficient of determination (adjusted *R*^2^) was also calculated. Two-tailed *P* values of <0.05 were considered statistically significant. All analyses were performed using STATA SE 11 data analysis and statistical software (Stata Corp LP, College Station, TX, USA).

## RESULTS

### Baseline characteristics of participants

Table 1 shows the characteristics of the participants according to HOMA-IR category by gender. The mean (SD) age was 60.8 (9.0) and 58.0 (8.7) years in men and women, respectively. Participants in higher HOMA-IR categories had higher HbA1c and FPG levels, both in men and women, and only men had lower 1,5-AG levels. Participants in higher HOMA-IR categories also had higher BMI and waist circumference, as well as lower HMW-adiponectin levels, both in men and women.

**Table 1.** Characteristics of the participants according to HOMA-IR values by gender

	HOMA-IR tertile			*P* value

	1st (low)	2nd	3rd (high)	
Men (*n* = 323)				
Number of participants	109	107	107	
HOMA-IR	<3.397	3.397–5.596	≥5.596	
HbA1c (NGSP; %), mean (SD)	5.47 (0.30)	5.43 (0.25)	5.63 (0.36)	<0.001
HbA1c (IFCC; mmol/mol), mean (SD)	36 (3)	36 (3)	38 (4)	<0.001
1,5-AG (µmol/L), mean (SD)	145.9 (46.9)	139.4 (45.0)	123.4 (40.9)	<0.001
FPG (mmol/L), mean (SD)	4.91 (0.34)	5.00 (0.35)	5.38 (0.50)	<0.001
IRI (pmol/L), mean (SD)	11.2 (3.1)	19.8 (3.0)	36.6 (12.4)	<0.001
Age (years), mean (SD)	61.1 (8.6)	60.0 (9.5)	61.3 (8.9)	0.495
Body mass index (kg/m^2^), mean (SD)	21.2 (2.2)	22.8 (2.1)	24.5 (2.3)	<0.001
Waist circumference (cm), mean (SD)	78.1 (6.6)	82.7 (6.0)	87.9 (7.7)	<0.001
Regular exercise, *n* (%)	70 (64.2%)	69 (64.5%)	66 (61.7%)	0.916
Current smoker, *n* (%)	15 (13.8%)	15 (14.0%)	5 (4.7%)	0.036
Current alcohol drinker, *n* (%)	86 (78.9%)	82 (76.6%)	82 (76.6%)	0.903
Chronic kidney disease, *n* (%)	9 (8.3%)	12 (11.2%)	15 (14.0%)	0.407
HMW-Adiponectin (µg/mL), median (IQR)	3.6 (2.6–5.2)	3.2 (2.0–4.7)	2.5 (1.6–3.7)	<0.001
Women (*n* = 760)				
Number of participants	254	255	251	
HOMA-IR	<3.126	3.126–4.819	≥4.819	
HbA1c (NGSP; %), mean (SD)	5.53 (0.31)	5.55 (0.27)	5.63 (0.29)	<0.001
HbA1c (IFCC; mmol/mol), mean (SD)	37 (3)	37 (3)	38 (3)	<0.001
1,5-AG (µmol/L), mean (SD)	105.6 (31.8)	107.1 (32.3)	109.5 (38.0)	0.444
FPG (mmol/L), mean (SD)	4.68 (0.34)	4.86 (0.33)	5.08 (0.40)	<0.001
IRI (pmol/L), mean (SD)	11.1 (2.7)	18.3 (2.4)	30.7 (10.1)	<0.001
Age (years), mean (SD)	57.0 (8.9)	58.4 (8.7)	58.5 (8.4)	0.094
Body mass index (kg/m^2^), mean (SD)	19.5 (2.1)	21.0 (2.3)	22.1 (2.6)	<0.001
Waist circumference (cm), mean (SD)	73.9 (7.3)	78.5 (7.3)	81.9 (7.6)	<0.001
Regular exercise, *n* (%)	141 (55.5%)	139 (54.5%)	124 (49.4%)	0.341
Current smoker, *n* (%)	7 (2.8%)	2 (0.8%)	6 (2.4%)	0.200
Current alcohol drinker, *n* (%)	107 (42.1%)	79 (31.0%)	89 (35.5%)	0.031
Chronic kidney disease, *n* (%)	18 (7.1%)	18 (7.1%)	20 (8.0%)	0.924
HMW-Adiponectin (µg/mL), median (IQR)	6.6 (4.6–8.8)	5.5 (3.9–7.8)	4.4 (3.1–6.1)	<0.001

1,5-AG, 1,5-anhydroglucitol; FPG, fasting plasma glucose; HMW-Adiponectin, high-molecular-weight adiponectin; HOMA-IR, homeostasis model assessment of insulin resistance; IFCC, International Federation of Clinical Chemistry and Laboratory Medicine; IQR, interquartile range; IRI, immunoreactive insulin; NGSP, National Glycohemoglobin Standardization Program; SD, standard deviation.

### Association between HOMA-IR values and markers of glycemic control

The association between HOMA-IR values and markers of glycemic control, such as HbA1c, 1,5-AG, and FPG levels, in the multivariate linear regression analysis are shown according to gender in Table 2 (men) and Table 3 (women). HbA1c and FPG levels were significantly higher in the highest tertile group of HOMA-IR values than in the lowest tertile group, both in men and women. 1,5-AG levels were significantly lower in the highest tertile group of HOMA-IR values than in the lowest tertile group in men but not in women. The association between HOMA-IR values and markers of glycemic control was unchanged after adjusting for potential confounders, including BMI, waist circumference, and HMW-adiponectin levels. We performed multiple linear regression analysis to estimate the association between HOMA-IR values and markers of glycemic control according to BMI and gender. The results showed that the absolute values of coefficient were larger in the group with high BMI than in the group with low BMI in men (eTable 1), which suggested a strong association between HOMA-IR and these glycemic control parameters; however, these findings were not clearly observed in women (eTable 2). We also performed multivariate logistic regression analysis to clarify the association between HOMA-IR values and any of the higher percentiles of HbA1c levels, lower percentiles of 1,5-AG levels, or higher percentiles of FPG levels, both in men (eTable 3) and women (eTable 4). Compared with the lowest tertile group of HOMA-IR values, the highest tertile group had significantly higher odds ratios for any of higher percentiles of HbA1c levels, lower percentiles of 1,5-AG levels, or higher percentiles of FPG levels, both in men and women, after adjusting for potential confounders.

**Table 2.** Associations between HOMA-IR values and markers of glycemic control in men (*n* = 323)

Dependent variables		Independent variables: HbA1c (mmol/mol)				Independent variables: 1,5-AG (µmol/L)				Independent variables: FPG (mmol/L)			
		
		Coefficient	95% CI	Standardized Coefficient	*P* value	Coefficient	95% CI	Standardized Coefficient	*P* value	Coefficient	95% CI	Standardized Coefficient	*P* value
Model 1													
HOMA-IR	1st (<3.397)	Reference				Reference				Reference			
	2nd (3.397–5.596)	−0.22	(−1.09, 0.65)	−0.03	0.621	−7.36	(−19.11, 4.39)	−0.08	0.219	0.10	(−0.01, 0.21)	0.10	0.064
	3rd (≥5.596)	1.77	(0.90, 2.64)	0.24	<0.001	−22.27	(−34.00, −10.53)	−0.23	<0.001	0.47	(0.36, 0.57)	0.49	<0.001
Age (10 years)		0.95	(0.55, 1.35)	0.25	<0.001	−7.86	(−13.23, −2.49)	−0.16	0.004	0.10	(0.05, 0.15)	0.20	<0.001
		Adjusted coefficient of determination (*R*^2^) = 0.12				Adjusted coefficient of determination (*R*^2^) = 0.06				Adjusted coefficient of determination (*R*^2^) = 0.23			
Model 2													
HOMA-IR	1st (<3.397)	Reference				Reference				Reference			
	2nd (3.397–5.596)	−0.33	(−1.24, 0.58)	−0.05	0.470	−7.04	(−19.16, 5.09)	−0.07	0.254	0.12	(0.01, 0.23)	0.12	0.034
	3rd (≥5.596)	1.65	(0.60, 2.70)	0.22	0.002	−18.62	(−32.60, −4.63)	−0.19	0.009	0.49	(0.36, 0.61)	0.51	<0.001
Age (10 years)		1.03	(0.59, 1.46)	0.26	<0.001	−6.42	(−12.25, −0.60)	−0.13	0.031	0.09	(0.04, 0.14)	0.18	0.001
Body mass index (kg/m^2^)		0.09	(−0.07, 0.26)	0.07	0.258	0.30	(−1.89, 2.50)	0.02	0.786	−0.01	(−0.03, 0.01)	−0.04	0.486
Regular exercise (yes)		−0.12	(−0.92, 0.67)	−0.02	0.758	4.20	(−6.36, 14.75)	0.04	0.434	−0.01	(−0.10, 0.09)	−0.01	0.912
Current smoking (yes)		1.02	(−0.18, 2.21)	0.09	0.094	30.94	(15.05, 46.82)	0.21	<0.001	−0.21	(−0.35, −0.06)	−0.14	0.005
Current alcohol drinking (yes)		−0.84	(−1.70, 0.02)	−0.10	0.056	−1.74	(−13.19, 9.72)	−0.02	0.766	0.07	(−0.03, 0.18)	0.07	0.161
Chronic kidney disease (yes)		0.08	(−1.08, 1.24)	0.01	0.895	−4.52	(−20.00, 10.96)	−0.03	0.566	−0.08	(−0.22, 0.06)	−0.06	0.240
HMW-Adiponectin (µg/mL)		0.32	(−0.31, 0.94)	0.06	0.319	3.57	(−4.74, 11.88)	0.05	0.399	0.02	(−0.06, 0.09)	0.03	0.613
		Adjusted coefficient of determination (*R*^2^) = 0.13				Adjusted coefficient of determination (*R*^2^) = 0.09				Adjusted coefficient of determination (*R*^2^) = 0.25			
Model 3													
HOMA-IR	1st (<3.397)	Reference				Reference				Reference			
	2nd (3.397–5.596)	−0.25	(−1.15, 0.66)	−0.03	0.595	−6.93	(−18.97, 5.11)	−0.07	0.258	0.12	(0.01, 0.23)	0.12	0.033
	3rd (≥5.596)	1.83	(0.79, 2.86)	0.25	0.001	−18.42	(−32.20, −4.64)	−0.19	0.009	0.49	(0.36, 0.61)	0.51	<0.001
Age (10 years)		1.00	(0.57, 1.44)	0.26	<0.001	−6.50	(−12.32, −0.69)	−0.13	0.028	0.09	(0.04, 0.14)	0.18	0.001
Waist circumference (10 cm)		0.13	(−0.41, 0.67)	0.03	0.635	0.83	(−6.33, 8.00)	0.01	0.819	−0.03	(−0.09, 0.04)	−0.04	0.441
Regular exercise (yes)		−0.11	(−0.90, 0.69)	−0.01	0.790	4.30	(−6.29, 14.89)	0.05	0.425	−0.01	(−0.10, 0.09)	−0.01	0.861
Current smoking (yes)		1.03	(−0.16, 2.23)	0.09	0.091	30.91	(15.00, 46.82)	0.21	<0.001	−0.21	(−0.35, −0.06)	−0.14	0.005
Current alcohol drinking (yes)		−0.84	(−1.70, 0.02)	−0.10	0.056	−1.76	(−13.22, 9.70)	−0.02	0.763	0.07	(−0.03, 0.18)	0.07	0.158
Chronic kidney disease (yes)		0.12	(−1.04, 1.29)	0.01	0.834	−4.38	(−19.82, 11.07)	−0.03	0.578	−0.09	(−0.23, 0.05)	−0.06	0.221
HMW-Adiponectin (µg/mL)		0.29	(−0.34, 0.91)	0.05	0.373	3.57	(−4.79, 11.93)	0.05	0.402	0.02	(−0.06, 0.09)	0.02	0.643
		Adjusted coefficient of determination (*R*^2^) = 0.13				Adjusted coefficient of determination (*R*^2^) = 0.09				Adjusted coefficient of determination (*R*^2^) = 0.25			

1,5-AG, 1,5-anhydroglucitol; CI, confidence interval; FPG, fasting plasma glucose; HMW-Adiponectin, high-molecular-weight adiponectin; HOMA-IR, homeostasis model assessment of insulin resistance.

Multivariate adjustment; Model 1: adjusted by age; Model 2: adjusted by age, body mass index, regular exercise (yes/no), current smoking (yes/no), current alcohol drinking (yes/no), chronic kidney disease (yes/no) and high-molecular-weight (HMW)-Adiponectin (log-transformed); Model 3: adjusted by age, waist circumference, regular exercise (yes/no), current smoking (yes/no), current alcohol drinking (yes/no), chronic kidney disease (yes/no) and HMW-Adiponectin (log-transformed).

**Table 3.** Associations between HOMA-IR values and markers of glycemic control in women (*n* = 760)

Dependent variables		Independent variables: HbA1c (mmol/mol)				Independent variables: 1,5-AG (µmol/L)				Independent variables: FPG (mmol/L)			
		
		Coefficient	95% CI	Standardized Coefficient	*P* value	Coefficient	95% CI	Standardized Coefficient	*P* value	Coefficient	95% CI	Standardized Coefficient	*P* value
Model 1													
HOMA-IR	1st (<3.126)	Reference				Reference				Reference			
	2nd (3.126–4.819)	0.18	(−0.36, 0.72)	0.03	0.516	1.90	(−4.03, 7.84)	0.03	0.529	0.16	(0.10, 0.22)	0.20	<0.001
	3rd (≥4.819)	1.00	(0.45, 1.54)	0.15	<0.001	4.35	(−1.61, 10.31)	0.06	0.152	0.38	(0.32, 0.44)	0.46	<0.001
Age (10 years)		0.88	(0.62, 1.13)	0.24	<0.001	−3.18	(−6.19, −0.59)	−0.09	0.018	0.11	(0.08, 0.14)	0.25	<0.001
		Adjusted coefficient of determination (*R*^2^) = 0.07				Adjusted coefficient of determination (*R*^2^) = 0.01				Adjusted coefficient of determination (*R*^2^) = 0.23			
Model 2													
HOMA-IR	1st (<3.126)	Reference				Reference				Reference			
	2nd (3.126–4.819)	0.01	(−0.55, 0.57)	0.001	0.974	0.83	(−5.34, 6.99)	0.01	0.792	0.16	(0.10, 0.22)	0.19	<0.001
	3rd (≥4.819)	0.80	(0.19, 1.41)	0.12	0.010	2.28	(−4.45, 9.00)	0.03	0.507	0.37	(0.31, 0.44)	0.45	<0.001
Age (10 years)		0.73	(0.44, 1.02)	0.20	<0.001	−3.06	(−6.28, 0.15)	−0.08	0.062	0.10	(0.07, 0.13)	0.23	<0.001
Body mass index (kg/m^2^)		0.07	(−0.02, 0.17)	0.06	0.130	0.96	(−0.11, 2.03)	0.07	0.077	0.01	(−0.004, 0.02)	0.04	0.286
Regular exercise (yes)		0.19	(−0.30, 0.68)	0.03	0.438	−2.19	(−7.58, 3.20)	−0.03	0.425	0.04	(−0.02, 0.09)	0.05	0.160
Current smoking (yes)		−1.12	(−2.73, 0.50)	−0.05	0.174	16.49	(−1.27, 34.25)	0.07	0.069	−0.01	(−0.19, 0.17)	−0.003	0.934
Current alcohol drinking (yes)		−0.62	(−1.08, −0.15)	−0.09	0.009	−2.01	(−7.12, 3.11)	−0.03	0.441	0.04	(−0.01, 0.09)	0.05	0.136
Chronic kidney disease (yes)		0.67	(−0.18, 1.52)	0.05	0.122	0.09	(−9.27, 9.44)	0.001	0.985	0.07	(−0.02, 0.17)	0.05	0.128
HMW-Adiponectin (µg/mL)		0.03	(−0.41, 0.47)	0.01	0.884	1.74	(−3.08, 6.57)	0.03	0.479	−0.001	(−0.05, 0.05)	−0.001	0.982
		Adjusted coefficient of determination (*R*^2^) = 0.08				Adjusted coefficient of determination (*R*^2^) = 0.01				Adjusted coefficient of determination (*R*^2^) = 0.23			
Model 3													
HOMA-IR	1st (<3.126)	Reference				Reference				Reference			
	2nd (3.126–4.819)	0.02	(−0.54, 0.58)	0.002	0.956	0.64	(−5.53, 6.80)	0.01	0.840	0.17	(0.11, 0.23)	0.20	<0.001
	3rd (≥4.819)	0.82	(0.21, 1.43)	0.12	0.008	2.09	(−4.59, 8.77)	0.03	0.539	0.39	(0.32, 0.45)	0.46	<0.001
Age (10 years)		0.70	(0.41, 1.00)	0.19	<0.001	−3.49	(−6.76, −0.22)	−0.09	0.036	0.10	(0.07, 0.14)	0.23	<0.001
Waist circumference (10 cm)		0.22	(−0.09, 0.53)	0.06	0.166	3.52	(0.09, 6.94)	0.08	0.044	0.0004	(−0.03, 0.04)	0.001	0.979
Regular exercise (yes)		0.20	(−0.29, 0.69)	0.03	0.425	−2.17	(−7.55, 3.21)	−0.03	0.428	0.04	(−0.01, 0.09)	0.05	0.144
Current smoking (yes)		−1.07	(−2.69, 0.54)	−0.05	0.192	17.11	(−0.64, 34.85)	0.07	0.059	−0.01	(−0.18, 0.17)	−0.002	0.956
Current alcohol drinking (yes)		−0.65	(−1.11, −0.18)	−0.10	0.007	−2.44	(−7.57, 2.69)	−0.03	0.350	0.04	(−0.01, 0.09)	0.05	0.141
Chronic kidney disease (yes)		0.68	(−0.17, 1.53)	0.05	0.119	0.18	(−9.17, 9.53)	0.001	0.971	0.07	(−0.02, 0.17)	0.05	0.130
HMW-Adiponectin (µg/mL)		0.03	(−0.41, 0.47)	0.01	0.883	1.98	(−2.87, 6.82)	0.03	0.424	−0.01	(−0.05, 0.04)	−0.01	0.811
		Adjusted coefficient of determination (*R*^2^) = 0.08				Adjusted coefficient of determination (*R*^2^) = 0.01				Adjusted coefficient of determination (*R*^2^) = 0.23			

1,5-AG, 1,5-anhydroglucitol; CI, confidence interval; FPG, fasting plasma glucose; HMW-Adiponectin, high-molecular-weight adiponectin; HOMA-IR, homeostasis model assessment of insulin resistance.

Multivariate adjustment; Model 1: adjusted by age; Model 2: adjusted by age, body mass index, regular exercise (yes/no), current smoking (yes/no), current alcohol drinking (yes/no), chronic kidney disease (yes/no) and high-molecular-weight (HMW)-Adiponectin (log-transformed); Model 3: adjusted by age, waist circumference, regular exercise (yes/no), current smoking (yes/no), current alcohol drinking (yes/no), chronic kidney disease (yes/no) and HMW-Adiponectin (log-transformed).

## DISCUSSION

This is the first report, to the best of our knowledge, to assess the relationship between HOMA-IR values and several indices of glucose metabolism, obtained at various time points, in Japanese subjects without diabetes or obesity. As a result, we found that HOMA-IR values were significantly associated with all indices of glucose metabolism in men, as well as with HbA1c or FPG levels in women.

HOMA-IR is generally considered an index of insulin resistance in the liver.^12^ Insulin suppresses the elevation of plasma glucose levels by promoting glucose uptake into cells and by inhibiting glucose release from the liver. However, when insulin resistance increases, the regulatory mechanism fails and blood glucose levels remain elevated.^14^^–^^16^ In the present study, our results indicate that increased insulin resistance further deteriorates glucose metabolism in patients with not only type 2 diabetes mellitus and obesity but also in those without diabetes or obesity. The relationship between insulin resistance and several indices of glucose metabolism was maintained after adjusting for confounding factors, such as BMI or waist circumference. Multivariate regression analysis in this study revealed that BMI and waist circumference were not correlated with the indices of glucose metabolism. However, in the multivariate regression model that excluded HOMA-IR as an independent variable, BMI (eTable 5) and waist circumference (eTable 6) maintained significant correlation with HbA1c and FPG levels. Therefore, these results indicated that insulin resistance might regulate glucose metabolism downstream of BMI and waist circumference.

The present study showed that HOMA-IR values were not significantly associated with 1,5-AG levels in women. 1,5-AG is monosaccharide excreted in the urine. Approximately, 99%–100% of the excreted 1,5-AG is reabsorbed in the renal tubules, and a constant level is maintained in subjects with normal glucose tolerance. When blood glucose levels reach the threshold at which urinary glucose appears, 1,5-AG levels decrease remarkably because 1,5-AG reabsorption is inhibited.^8^^,^^17^^,^^18^ In other words, 1,5-AG levels do not change if blood glucose levels do not reach the urinary glucose excretion threshold. Considering these mechanisms, it is suspected that most of the participants, especially women, had normal glucose tolerance, although participants both with normal glucose tolerance and mild glucose intolerance were included in the present study. In male participants, HOMA-IR values were weakly correlated with 1,5-AG levels compared to the correlations of HOMA-IR values with HbA1c and FPG levels. Thus, it is possible that the suspected high prevalence of normal glucose tolerance influenced this result.

A cohort study of the general Japanese population revealed that metabolic syndrome increased the risk of onset of type 2 diabetes mellitus, suggesting that insulin resistance contributed to the onset of type 2 diabetes mellitus in the Japanese population.^19^ Another cohort study of the general Japanese population showed that both a decrease in insulin secretion ability and an increase in insulin resistance contributed to the onset of type 2 diabetes mellitus.^20^ In the present study, a correlation between an index of insulin resistance and several indices of glucose metabolism was observed in Japanese subjects without obesity and with low insulin resistance. These findings suggest that insulin resistance mainly contributed to the onset of type 2 diabetes mellitus in Japanese subjects. Lifestyle interventions, such as healthy diet and regular exercise, have been shown to be effective for the improvement of insulin resistance but not impaired insulin secretion capacity.^21^ Thus, we recommend lifestyle interventions for the prevention of onset of type 2 diabetes mellitus not only in obese subjects but also in non-obese subjects. In the present study, we also found that the association between HOMA-IR values and each marker of glycemic control was stronger in subjects with high BMI than in those with low BMI. Therefore, even in non-obese (BMI <30 kg/m^2^) Asians, we suggest that the impact of lifestyle intervention on the onset of type 2 diabetes mellitus is larger among subjects with high BMI than among those with low BMI when impaired insulin secretion capacity is suspected.

This study has several limitations. At first, we used HOMA-IR, which is an indirect index for the evaluation of insulin resistance. Although the glucose clamp technique is necessary for direct evaluation,^22^ we were unable to apply this test in our subjects. However, a previous study reported that the use of HOMA-IR was appropriate to assess insulin sensitivity in subjects without diabetes.^23^ Second, we used a self-reported questionnaire in the present study; thus, recall bias might have affected the evaluation of physical activity. Finally, we could not evaluate postprandial hyperglycemia accurately in this study because we did not measure the blood glucose afterload. We used HbA1c levels as the diagnostic criteria of diabetes in this study; thus, subjects having marked postprandial hyperglycemia were excluded.

In conclusion, the present study showed that HOMA-IR values were significantly associated with several indices of glucose metabolism in Japanese subjects without diabetes or obesity and that insulin resistance prescribed glucose metabolism downstream of BMI or waist circumference. These findings suggest that insulin resistance may mainly influence glycemic control even in non-diabetic subjects without obesity.

## ONLINE ONLY MATERIALS

eTable 1. Associations between HOMA-IR values and markers of glycemic control divided by median BMI in men (*n* = 323).

eTable 2. Associations between HOMA-IR values and markers of glycemic control divided by median BMI in women (*n* = 760).

eTable 3. Associations between HOMA-IR values and higher percentile of HbA1c or FPG or lower percentile of 1,5-AG.

eTable 4. Associations between HOMA-IR values and higher percentile of HbA1c or FPG or lower percentile of 1,5-AG.

eTable 5. Associations between BMI and markers of glycemic control in multivariate regression model that excluded HOMA-IR.

eTable 6. Associations between waist circumference and markers of glycemic control in multivariate regression model that excluded HOMA-IR.

Abstract in Japanese.
