# Supplementary material for: HOMA-IR Values are Associated With Glycemic Control in Japanese Subjects Without Diabetes or Obesity: The KOBE Study
Source: J Epidemiol. 2015 Jun 5;25(6):407–14. doi: 10.2188/jea.JE20140172 (PMC4444494; doi:10.2188/jea.JE20140172)
Supplement: eTable 1. [file je-25-407-s001.pdf]

eTable 1. Associations between HOMA-IR values and markers of glycemic control divided by median BMI in men (n=323)

Dependent variables		Independent variables: HbA1c (mmol/mol)				Independent variables: 1,5-AG (μmol/L)				Independent variables: FPG (mmol/L)			
		Coefficient	95% CI	Standardized Coefficient	*P* value	Coefficient	95% CI	Standardized Coefficient	*P* value	Coefficient	95% CI	Standardized Coefficient	*P* value
**High-BMI Group (22.7−29.8, *n* =161)**													
HOMA-IR	1st (<4.742)	Reference				Reference				Reference			
	2nd (4.742−7.491)	1.84	(0.59, 3.08)	0.24	0.004	−16.22	(−31.84, −0.61)	−0.18	0.042	0.27	(0.11, 0.42)	0.26	0.001
	3rd (≥7.491)	3.23	(1.98, 4.48)	0.42	<0.001	−24.62	(−40.33, −8.91)	−0.27	0.002	0.59	(0.44, 0.75)	0.58	<0.001
Age (10 years)		1.00	(0.45, 1.55)	0.26	<0.001	−9.81	(−16.71, −2.90)	−0.21	0.006	0.11	(0.04, 0.18)	0.22	0.001
		Adjusted coefficient of determination (R^2) = 0.19				Adjusted coefficient of determination (R^2) = 0.09				Adjusted coefficient of determination (R^2) = 0.30			
**Low-BMI Group (15.8−22.6, *n* =162)**													
HOMA-IR	1st (<2.694)	Reference				Reference				Reference			
	2nd (2.694−4.021)	0.40	(−0.80, 1.59)	0.06	0.515	−1.64	(−19.47, 16.19)	−0.02	0.856	0.24	(0.10, 0.38)	0.28	0.001
	3rd (≥4.021)	0.16	(−1.04, 1.36)	0.02	0.789	−9.35	(−27.19, 8.49)	−0.09	0.302	0.27	(0.13, 0.41)	0.32	<0.001
Age (10 years)		0.94	(0.37, 1.50)	0.25	0.001	−5.88	(−14.32, 2.57)	−0.11	0.171	0.09	(0.02, 0.16)	0.20	0.008
		Adjusted coefficient of determination (R^2) = 0.05				Adjusted coefficient of determination (R^2) = 0.00				Adjusted coefficient of determination (R^2) = 0.11			

1,5-AG, 1,5-anhydroglucitol; BMI, body mass index; CI, confidence interval; FPG, fasting plasma glucose; HOMA-IR, homeostasis model assessment of insulin resistance.

Participants were divided into two groups by the median body mass index (BMI) level. Association between HOMA-IR and each marker of glycemic control was adjusted by age.
