# Supplementary material for: HOMA-IR Values are Associated With Glycemic Control in Japanese Subjects Without Diabetes or Obesity: The KOBE Study
Source: J Epidemiol. 2015 Jun 5;25(6):407–14. doi: 10.2188/jea.JE20140172 (PMC4444494; doi:10.2188/jea.JE20140172)
Supplement: eTable 2. [file je-25-407-s002.pdf]

eTable 2. Associations between HOMA-IR values and markers of glycemic control divided by median BMI in women (*n* =760)

Dependent variables		Independent variables: HbA1c (mmol/mol)				Independent variables: 1,5-AG (μmol/L)				Independent variables: FPG (mmol/L)			
		Coefficient	95% CI	Standardized Coefficient	*P* value	Coefficient	95% CI	Standardized Coefficient	*P* value	Coefficient	95% CI	Standardized Coefficient	*P* value
**High-BMI Group (20.56−29.6, *n* =380)**													
HOMA-IR	1st (<3.836)	Reference				Reference				Reference			
	2nd (3.836−5.475)	0.79	(0.03, 1.56)	0.12	0.042	−0.33	(−8.45, 7.80)	−0.004	0.937	0.18	(0.09, 0.26)	0.21	<0.001
	3rd (≥5.475)	0.74	(−0.03, 1.51)	0.11	0.058	−1.04	(−9.19, 7.11)	−0.01	0.802	0.35	(0.26, 0.43)	0.42	<0.001
Age (10 years)		0.97	(0.60, 1.34)	0.26	<0.001	−3.36	(−7.26, 0.54)	−0.09	0.091	0.09	(0.05, 0.13)	0.20	<0.001
		Adjusted coefficient of determination (R^2) = 0.08				Adjusted coefficient of determination (R^2) = −0.000				Adjusted coefficient of determination (R^2) = 0.17			
**Low-BMI Group (13.6−20.55, *n* =380)**													
HOMA-IR	1st (<2.643)	Reference				Reference				Reference			
	2nd (2.643−3.897)	0.29	(−0.49, 1.06)	0.04	0.463	1.89	(−6.81, 10.59)	0.03	0.670	0.17	(0.09, 0.25)	0.21	<0.001
	3rd (≥3.897)	0.51	(−0.27, 1.28)	0.07	0.202	4.85	(−3.88, 13.58)	0.06	0.276	0.37	(0.28, 0.45)	0.45	<0.001
Age (10 years)		0.71	(0.34, 1.07)	0.19	<0.001	−3.96	(−8.06, 0.14)	−0.10	0.058	0.12	(0.08, 0.16)	0.28	<0.001
		Adjusted coefficient of determination (R^2) = 0.04				Adjusted coefficient of determination (R^2) = 0.004				Adjusted coefficient of determination (R^2) = 0.24			

1,5-AG, 1,5-anhydroglucitol; BMI, body mass index; CI, confidence interval; FPG, fasting plasma glucose; HOMA-IR, homeostasis model assessment of insulin resistance.

Participants were divided into two groups by the median body mass index (BMI) level. Association between HOMA-IR and each marker of glycemic control was adjusted by age.
