# Supplementary material for: HOMA-IR Values are Associated With Glycemic Control in Japanese Subjects Without Diabetes or Obesity: The KOBE Study
Source: J Epidemiol. 2015 Jun 5;25(6):407–14. doi: 10.2188/jea.JE20140172 (PMC4444494; doi:10.2188/jea.JE20140172)
Supplement: eTable 3. [file je-25-407-s003.pdf]

eTable 3. Associations between HOMA-IR values and higher percentiles of HbA1c or FPG, or lower percentiles of 1,5-AG divided by gender using logistic regression analysis in men (*n* =323)

Dependent variables		Independent variables: 10th percentile of HbA1c, 1,5-AG, or FPG			Independent variables: 20th percentile of HbA1c, 1,5-AG, or FPG		
		Odds ratio	95% CI	*P* value	Odds ratio	95% CI	*P* value
Model 1							
HOMA-IR	1st (<3.397)	Reference			Reference		
	2nd (3.397−5.596)	1.20	(0.48, 2.98)	0.700	0.81	(0.44, 1.50)	0.503
	3rd (≥5.596)	8.09	(3.74, 17.53)	<0.001	3.94	(2.20, 7.05)	<0.001
Age (10 years)		2.01	(1.36, 2.97)	<0.001	1.77	(1.32, 2.37)	<0.001
Model 2							
HOMA-IR	1st (<3.397)	Reference			Reference		
	2nd (3.397−5.596)	1.28	(0.50, 3.29)	0.613	0.84	(0.44, 1.61)	0.604
	3rd (≥5.596)	9.29	(3.59, 24.01)	<0.001	4.50	(2.17, 9.31)	<0.001
Age (10 years)		2.01	(1.31, 3.09)	0.001	1.90	(1.36, 2.64)	<0.001
Body mass index (kg/m^2)		0.95	(0.83, 1.10)	0.501	1.00	(0.89, 1.12)	0.995
Regular exercise (yes)		0.95	(0.48, 1.90)	0.889	0.60	(0.34, 1.05)	0.072
Current smoking (yes)		1.19	(0.36, 3.97)	0.775	1.00	(0.42, 2.41)	0.997
Current alcohol drinking (yes)		0.85	(0.42, 1.74)	0.661	0.66	(0.37, 1.18)	0.163
Chronic kidney disease (yes)		1.20	(0.49, 2.98)	0.688	1.36	(0.60, 3.05)	0.458
HMW-Adiponectin (μg/mL)		0.93	(0.54, 1.58)	0.787	1.38	(0.88, 2.15)	0.156
Model 3							
HOMA-IR	1st (<3.397)	Reference			Reference		
	2nd (3.397−5.596)	1.16	(0.45, 2.97)	0.759	0.86	(0.45, 1.63)	0.637
	3rd (≥5.596)	7.59	(3.03, 18.99)	<0.001	4.65	(2.26, 9.56)	<0.001
Age (10 years)		2.02	(1.32, 3.11)	0.001	1.90	(1.36, 2.65)	<0.001
Waist circumference (10 cm)		1.06	(0.67, 1.66)	0.808	0.96	(0.66, 1.40)	0.847
Regular exercise (yes)		0.97	(0.48, 1.95)	0.936	0.59	(0.34, 1.04)	0.070
Current smoking (yes)		1.12	(0.34, 3.75)	0.850	1.01	(0.42, 2.43)	0.982
Current alcohol drinking (yes)		0.85	(0.42, 1.73)	0.653	0.66	(0.37, 1.19)	0.164
Chronic kidney disease (yes)		1.15	(0.47, 2.85)	0.758	1.36	(0.61, 3.05)	0.455
HMW-Adiponectin (μg/mL)		0.97	(0.56, 1.66)	0.911	1.36	(0.87, 2.13)	0.172

1,5-AG, 1,5-anhydroglucitol; CI, confidence interval; FPG, fasting plasma glucose; HMW-Adiponectin, high molecular weight adiponectin; HOMA-IR, homeostasis model assessment of insulin resistance.

Multivariate adjustment; Model 1: adjusted by age; Model 2: adjusted by age, body mass index, regular exercise (yes/no), current smoking (yes/no), current alcohol drinking (yes/no), chronic kidney disease (yes/no) and high-molecular-weight (HMW)-Adiponectin (log-transformed); Model 3: adjusted by age, waist circumference, regular exercise (yes/no), current smoking (yes/no), current alcohol drinking (yes/no), chronic kidney disease (yes/no) and HMW-Adiponectin (log-transformed)
