# Supplementary material for: HOMA-IR Values are Associated With Glycemic Control in Japanese Subjects Without Diabetes or Obesity: The KOBE Study
Source: J Epidemiol. 2015 Jun 5;25(6):407–14. doi: 10.2188/jea.JE20140172 (PMC4444494; doi:10.2188/jea.JE20140172)
Supplement: eTable 4. [file je-25-407-s004.pdf]

eTable 4. Associations between HOMA-IR values and higher percentile of HbA1c or FPG, or lower percentile of 1,5-AG divided by gender using logistic regression analysis in women (*n* =760)

Dependent variables		Independent variables: 10th percentile of HbA1c, 1,5-AG, or FPG			Independent variables: 20th percentile of HbA1c, 1,5-AG, or FPG		
		Odds ratio	95% CI	*P* value	Odds ratio	95% CI	*P* value
Model 1							
HOMA-IR	1st (<3.126)	Reference			Reference		
	2nd (3.126−4.819)	1.03	(0.66, 1.61)	0.900	0.97	(0.67, 1.40)	0.853
	3rd (≥4.819)	2.00	(1.31, 3.04)	0.001	1.81	(1.26, 2.60)	0.001
Age (10 years)		1.70	(1.38, 2.11)	<0.001	1.58	(1.33, 1.89)	<0.001
Model 2							
HOMA-IR	1st (<3.126)	Reference			Reference		
	2nd (3.126−4.819)	1.13	(0.71, 1.79)	0.619	1.03	(0.70, 1.51)	0.896
	3rd (≥4.819)	2.35	(1.45, 3.78)	<0.001	2.04	(1.35, 3.09)	0.001
Age (10 years)		1.56	(1.23, 1.99)	<0.001	1.48	(1.21, 1.82)	<0.001
Body mass index (kg/m^2)		0.95	(0.88, 1.03)	0.235	0.95	(0.89, 1.02)	0.139
Regular exercise (yes)		1.37	(0.93, 2.01)	0.116	1.28	(0.92, 1.78)	0.146
Current smoking (yes)		0.37	(0.05, 2.90)	0.341	0.50	(0.13, 1.85)	0.297
Current alcohol drinking (yes)		1.18	(0.82, 1.70)	0.374	1.03	(0.75, 1.41)	0.877
Chronic kidney disease (yes)		1.70	(0.94, 3.07)	0.077	1.64	(0.93, 2.89)	0.089
HMW-Adiponectin (μg/mL)		0.99	(0.70, 1.40)	0.969	0.92	(0.68, 1.23)	0.562
Model 3							
HOMA-IR	1st (<3.126)	Reference			Reference		
	2nd (3.126−4.819)	1.22	(0.76, 1.96)	0.406	1.02	(0.70, 1.50)	0.913
	3rd (≥4.819)	2.67	(1.65, 4.34)	<0.001	2.01	(1.33, 3.04)	0.001
Age (10 years)		1.66	(1.29, 2.12)	<0.001	1.51	(1.23, 1.85)	<0.001
Waist circumference (10 cm)		0.71	(0.55, 0.92)	0.008	0.87	(0.70, 1.07)	0.188
Regular exercise (yes)		1.39	(0.94, 2.05)	0.098	1.28	(0.92, 1.78)	0.150
Current smoking (yes)		0.35	(0.04, 2.82)	0.326	0.48	(0.13, 1.79)	0.277
Current alcohol drinking (yes)		1.23	(0.85, 1.77)	0.278	1.04	(0.76, 1.43)	0.793
Chronic kidney disease (yes)		1.69	(0.94, 3.06)	0.081	1.63	(0.92, 2.87)	0.092
HMW-Adiponectin (μg/mL)		0.93	(0.66, 1.31)	0.687	0.92	(0.68, 1.24)	0.572

1,5-AG, 1,5-anhydroglucitol; CI, confidence interval; FPG, fasting plasma glucose; HMW-Adiponectin, high molecular weight adiponectin; HOMA-IR, homeostasis model assessment of insulin resistance.

Multivariate adjustment; Model 1: adjusted by age; Model 2: adjusted by age, body mass index, regular exercise (yes/no), current smoking (yes/no), current alcohol drinking (yes/no), chronic kidney disease (yes/no) and high-molecular-weight (HMW)-Adiponectin (log-transformed); Model 3: adjusted by age, waist circumference, regular exercise (yes/no), current smoking (yes/no), current alcohol drinking (yes/no), chronic kidney disease (yes/no) and HMW-Adiponectin (log-transformed)
