# Supplementary material for: HOMA-IR Values are Associated With Glycemic Control in Japanese Subjects Without Diabetes or Obesity: The KOBE Study
Source: J Epidemiol. 2015 Jun 5;25(6):407–14. doi: 10.2188/jea.JE20140172 (PMC4444494; doi:10.2188/jea.JE20140172)
Supplement: eTable 5. [file je-25-407-s005.pdf]

eTable 5. Associations between BMI and markers of glycemic control in multivariate regression model that excluded HOMA-IR

Dependent variables	Independent variables: HbA1c (mmol/mol)				Independent variables: 1,5-AG (μmol/L)				Independent variables: FPG (mmol/L)			
	Coefficient	95% CI	Standardized Coefficient	*P* value	Coefficient	95% CI	Standardized Coefficient	*P* value	Coefficient	95% CI	Standardized Coefficient	*P* value
Men (*n* =323)												
Body mass index (kg/m^2)	0.22	(0.07, 0.37)	0.16	0.004	−1.11	(−3.05, 0.82)	−0.06	0.259	0.03	(0.01, 0.05)	0.17	0.002
Age (10 years)	1.10	(0.65, 1.54)	0.28	<0.001	−6.81	(−12.66, −0.96)	−0.14	0.023	0.10	(0.04, 0.16)	0.20	0.001
Regular exercise (yes)	−0.19	(−1.00, 0.62)	−0.03	0.644	4.59	(−6.04, 15.22)	0.05	0.396	−0.02	(−0.12, 0.09)	−0.02	0.737
Current smoking (yes)	0.64	(−0.56, 1.85)	0.06	0.296	34.20	(18.39, 50.01)	0.24	<0.001	−0.30	(−0.46, −0.14)	−0.21	<0.001
Current alcohol drinking (yes)	−0.88	(−1.76, 0.00)	−0.11	0.051	−1.20	(−12.74, 10.34)	−0.01	0.838	0.06	(−0.05, 0.17)	0.06	0.295
Chronic kidney disease (yes)	0.12	(−1.07, 1.31)	0.01	0.843	−5.18	(−20.77, 10.41)	−0.04	0.514	−0.07	(−0.22, 0.09)	−0.05	0.385
HMW-Adiponectin (μg/mL)	0.11	(−0.52, 0.74)	0.02	0.721	5.56	(−2.68, 13.80)	0.08	0.185	−0.03	(−0.12, 0.05)	−0.05	0.404
	Adjusted coefficient of determination (R^2) = 0.09				Adjusted coefficient of determination (R^2) = 0.08				Adjusted coefficient of determination (R^2) = 0.11			
Women (*n* =760)												
Body mass index (kg/m^2)	0.12	(0.03, 0.21)	0.09	0.012	1.09	(−6.76, −0.22)	0.08	0.033	0.03	(0.02, 0.04)	0.17	<0.001
Age (10 years)	0.77	(0.48, 1.06)	0.21	<0.001	−2.93	(0.09, 6.94)	−0.07	0.071	0.12	(0.09, 0.16)	0.27	<0.001
Regular exercise (yes)	0.11	(−0.37, 0.60)	0.02	0.648	−2.42	(−7.76, 2.93)	−0.04	0.375	0.00	(−0.06, 0.06)	0.00	0.941
Current smoking (yes)	−1.13	(−2.75, 0.49)	−0.05	0.170	16.32	(−1.39, 34.02)	0.07	0.071	−0.04	(−0.23, 0.15)	−0.01	0.678
Current alcohol drinking (yes)	−0.60	(−1.07, −0.14)	−0.09	0.011	−2.02	(−7.12, 3.07)	−0.03	0.436	0.04	(−0.02, 0.09)	0.04	0.212
Chronic kidney disease (yes)	0.68	(−0.18, 1.53)	0.05	0.119	0.09	(−9.26, 9.43)	0.00	0.985	0.07	(−0.03, 0.17)	0.05	0.158
HMW-Adiponectin (μg/mL)	−0.10	(−0.53, 0.33)	−0.02	0.636	1.38	(−3.32, 6.09)	0.02	0.564	−0.06	(−0.12, −0.01)	−0.08	0.025
	Adjusted coefficient of determination (R^2) = 0.08				Adjusted coefficient of determination (R^2) = 0.01				Adjusted coefficient of determination (R^2) = 0.12			

1,5-AG, 1,5-anhydroglucitol; BMI, body mass index; CI, confience interval; FPG, fasting plasma glucose; HOMA-IR, homeostasis model assessment of insulin resistance.

Multivariate adjustment; adjusted by age, regular exercise (yes/no), current smoking (yes/no), current alcohol drinking (yes/no), chronic kidney disease (yes/no) and high-molecular-weight (HMW)-Adiponectin (log-transformed)
