# Supplementary material for: HOMA-IR Values are Associated With Glycemic Control in Japanese Subjects Without Diabetes or Obesity: The KOBE Study
Source: J Epidemiol. 2015 Jun 5;25(6):407–14. doi: 10.2188/jea.JE20140172 (PMC4444494; doi:10.2188/jea.JE20140172)
Supplement: eTable 6. [file je-25-407-s006.pdf]

eTable 6. Associations between waist circumference and markers of glycemic control in multivariate regression model that excluded HOMA-IR

Dependent variables	Independent variables: HbA1c (mmol/mol)				Independent variables: 1,5-AG (μmol/L)				Independent variables: FPG (mmol/L)			
	Coefficient	95% CI	Standardized Coefficient	*P* value	Coefficient	95% CI	Standardized Coefficient	*P* value	Coefficient	95% CI	Standardized Coefficient	*P* value
Men (*n* =323)												
Waist circumference (10 cm)	0.57	(0.08, 1.06)	0.13	0.023	−3.58	(−9.99, 2.83)	−0.06	0.273	0.09	(0.03, 0.15)	0.16	0.005
Age (10 years)	1.04	(0.59, 1.49)	0.27	<0.001	−6.50	(−12.34, −0.65)	−0.13	0.030	0.09	(0.04, 0.15)	0.18	0.002
Regular exercise (yes)	−0.12	(−0.94, 0.70)	−0.02	0.767	4.15	(−6.51, 14.82)	0.04	0.444	−0.01	(−0.11, 0.10)	−0.01	0.900
Current smoking (yes)	0.60	(−0.61, 1.81)	0.05	0.332	34.44	(18.62, 50.25)	0.24	<0.001	−0.31	(−0.46, −0.15)	−0.21	<0.001
Current alcohol drinking (yes)	−0.90	(−1.79, −0.01)	−0.11	0.047	−1.09	(−12.63, 10.44)	−0.01	0.852	0.06	(−0.06, 0.17)	0.05	0.318
Chronic kidney disease (yes)	0.24	(−0.95, 1.44)	0.02	0.689	−5.75	(−21.28, 9.78)	−0.04	0.467	−0.05	(−0.21, 0.10)	−0.04	0.502
HMW-Adiponectin (μg/mL)	0.07	(−0.57, 0.71)	0.01	0.822	5.48	(−2.83, 13.79)	0.07	0.195	−0.03	(−0.12, 0.05)	−0.05	0.411
	Adjusted coefficient of determination (R^2) = 0.08				Adjusted coefficient of determination (R^2) = 0.08				Adjusted coefficient of determination (R^2) = 0.10			
Women (*n* =760)												
Waist circumference (10 cm)	0.35	(0.05, 0.64)	0.09	0.020	3.87	(0.65, 7.09)	0.09	0.019	0.07	(0.03, 0.10)	0.14	<0.001
Age (10 years)	0.74	(0.44, 1.04)	0.20	<0.001	−3.41	(−6.66, −0.15)	−0.09	0.040	0.12	(0.08, 0.15)	0.26	<0.001
Regular exercise (yes)	0.12	(−0.37, 0.61)	0.02	0.634	−2.37	(−7.71, 2.96)	−0.03	0.383	0.00	(−0.05, 0.06)	0.00	0.909
Current smoking (yes)	−1.06	(−2.68, 0.56)	−0.05	0.198	17.03	(−0.67, 34.73)	0.07	0.059	−0.03	(−0.22, 0.17)	−0.01	0.785
Current alcohol drinking (yes)	−0.65	(−1.11, −0.18)	−0.10	0.007	−2.49	(−7.59, 2.61)	−0.04	0.339	0.03	(−0.03, 0.08)	0.03	0.353
Chronic kidney disease (yes)	0.69	(−0.17, 1.54)	0.06	0.115	0.19	(−9.15, 9.53)	0.00	0.968	0.07	(−0.03, 0.18)	0.05	0.154
HMW-Adiponectin (μg/mL)	−0.11	(−0.54, 0.32)	−0.02	0.625	1.64	(−3.09, 6.37)	0.03	0.496	−0.07	(−0.12, −0.01)	−0.09	0.013
	Adjusted coefficient of determination (R^2) = 0.07				Adjusted coefficient of determination (R^2) = 0.01				Adjusted coefficient of determination (R^2) = 0.11			

1,5-AG, 1,5-anhydroglucitol; BMI, body mass index; CI, confience interval; FPG, fasting plasma glucose; HOMA-IR, homeostasis model assessment of insulin resistance.

Multivariate adjustment; adjusted by age, regular exercise (yes/no), current smoking (yes/no), current alcohol drinking (yes/no), chronic kidney disease (yes/no) and high-molecular-weight (HMW)-Adiponectin (log-transformed)
