# Supplementary material for: HOMA-IR Values are Associated With Glycemic Control in Japanese Subjects Without Diabetes or Obesity: The KOBE Study
Source: J Epidemiol. 2015 Jun 5;25(6):407–14. doi: 10.2188/jea.JE20140172 (PMC4444494; doi:10.2188/jea.JE20140172)
Supplement: Abstract in Japanese. [file je-25-407-s007.pdf]

肥満・糖尿病を有さない日本人において HOMA-IR は血糖コントロールと関連する：神戸研究

平田 匠[1)]、東山 綾[1,2)]、久保田 芳美[1,3)]、西村 邦宏[1,2)]、杉山 大典[1,4)]、門田 文[1,5)]、西田 陽子[1)]、今野 弘規[1,6)]、西川 智文[1,7)]、宮松 直美[1,8)]、宮本 恵宏[1,2)]、岡村 智教[1,4)]

1) 公益財団法人 先端医療振興財団
2) 国立循環器病研究センター 予防医学・疫学情報部
3) 兵庫医科大学 環境予防医学
4) 慶應義塾大学医学部 衛生学公衆衛生学
5) 滋賀医科大学 アジア疫学研究センター
6) 大阪大学大学院医学系研究科 公衆衛生学
7) 京都光華女子大学 健康科学部
8) 滋賀医科大学 臨床看護学講座

【背景】これまで肥満ではなく糖尿病も有さない健常者においてインスリン抵抗性は 2 型糖尿病の危険因子であることが報告されているが、肥満度の低い日本人集団では検証されていない。そこで肥満・糖尿病を有さない日本人において、インスリン抵抗性が血糖コントロール指標と関連するかについて検討した。
【方法】都市住民の健常者 1,083 名（男性 323 名、女性 760 名、平均 BMI 21.5 kg/m^2）のデータを用いて断面研究を行った。交絡因子調整後におけるインスリン抵抗性の指標（HOMA-IR）と血糖コントロール指標（HbA1c、1,5-AG、空腹時血糖）の関連について、多変量回帰分析で検討した。
【結果】交絡因子調整後の結果において、男女とも HOMA-IR 最低三分位群と比較し、最高三分位群で HbA1c および空腹時血糖が有意に高かった（男性: HbA1c; $\beta = 1.83$, $P = 0.001$, 空腹時血糖; $\beta = 0.49$, $P < 0.001$、女性: HbA1c; $\beta = 0.82$, $P = 0.008$, 空腹時血糖; $\beta = 0.39$, $P < 0.001$）。また男性でのみ、HOMA-IR 最低三分位群と比較し、最高三分位群で 1,5-AG が有意に低かった（$\beta = -18.42$, $P = 0.009$）。
【結論】肥満・糖尿病を有さない日本人において HOMA-IR は血糖コントロール指標と関連しており、肥満や糖尿病を有さなくてもインスリン抵抗性が血糖コントロールに影響を及ぼしている可能性が示唆された。

キーワード：HOMA-IR、血糖コントロール、疫学
